# Preventing breast milk HIV transmission using broadly neutralizing monoclonal antibodies: One size does not fit all

**DOI:** 10.1002/iid3.1216

**Published:** 2024-03-27

**Authors:** Philippe Van de Perre, Gabriella Scarlatti, Penny L. Moore, Jean‐Pierre Molès, Nicolas Nagot, Thorkild Tylleskär, Glenda Gray, Ameena Goga

**Affiliations:** ^1^ Pathogenesis and Control of Chronic and Emerging Infections, INSERM, Etablissement Français du Sang, CHU Montpellier University of Montpellier Montpellier France; ^2^ Viral Evolution and Transmission Unit IRCCS Ospedale San Raffaele Milan Italy; ^3^ MRC Antibody Immunity Research Unit, School of Pathology University of the Witwatersrand Johannesburg South Africa; ^4^ National Institute for Communicable Diseases (NICD) of the National Health Laboratory Service (NHLS) Johannesburg South Africa; ^5^ Department of Global Public Health and Primary Care, Centre for International Health University of Bergen Bergen Norway; ^6^ South African Medical Research Council Cape Town South Africa; ^7^ Department of Paediatrics and Child Health University of Pretoria Pretoria South Africa

**Keywords:** antibodies molecules, human animals, viral/retroviral infections

## Abstract

- Passive immunoprophylaxis with broadly neutralizing monoclonal antibodies (bNAbs) could be a game changer in the prevention of human immunodeficiency virus (HIV) acquisition.
- The prevailing view is that available resources should be focused on identifying a fixed combination of at least three bNAbs for universal use in therapeutic and preventive protocols, regardless of target populations or routes of transmission.
- HIV transmission through breastfeeding is unique: it involves free viral particles and cell‐associated virus from breast milk and, in the case of acute/recent maternal infection, a viral population with restricted Env diversity.
- HIV transmission through breastfeeding in high incidence/prevalence areas could potentially be eliminated by subcutaneous administration to all newborns of one or two long‐acting bNAbs with extended breadth, high potency, and effector properties (ADCC, phagocytosis) against circulating HIV strains.

## 1 INTRODUCTION

Broadly neutralizing monoclonal antibodies (bNAbs) against human immunodeficiency virus (HIV) have been developed at an impressive rate in recent years.^1^ We now have a portfolio of more than 17 bNAbs for potential therapeutic and/or preventive clinical development, targeting the CD4 binding site, V1V2 glycans, V3 glycans, membrane proximal external region, gp120‐gp41 interface, or silent face of the HIV envelope.^1^ The Fc region of several bNAbs has been modified by substitution of two amino acids, methionine and asparagine, at position M428L/N434S, referred to as the “LS” mutation, to increase the binding affinity for the neonatal Fc receptor (FcRn), resulting in improved protection against SHIV infection in nonhuman primates,^2^ extending the half‐life to up to approximately 3 months^3^ and allowing administration of small volumes by subcutaneous route, particularly in neonates and young children.^4^ Versions of some bNAbs with more favorable biological properties or manufacturability, such as CAP256V2LS^5^ or VRC07‐523LS,^6^ have also been further engineered. The potency and breadth of several of these bNAbs against a wide range of HIV strains have been well characterized in vitro and a limited number of them are currently under clinical evaluation in children, including VRC01, PGT121.414, PGDM1400, VRC07‐523LS, 10‐1074, and CAP256V2 and their long‐acting variants. Two of these antibodies, VRC01LS and 10‐1074, have recently been evaluated as potential HIV therapies in children and have been associated with HIV control in some children living with HIV who have discontinued antiretroviral therapy.^7^

## 2 PREVENTION OF HIV ACQUISITION: COCKTAIL VERSUS SINGLE/DUAL bNAbs REGIMEN?

The dominant view on the use of bNAbs to prevent HIV transmission is that a single predefined cocktail of preferably three or more bNAbs or a tri‐ or quadrivalent product (combining Fabs from different bNAbs on a single antibody molecule) should be developed and tested. It is postulated that such an approach will protect against HIV in all parts of the world, regardless of the route of transmission, the local circulation of HIV strains, or the characteristics of the target population.^8^ This position is largely based on observations from the Antibody‐Mediated Prevention (AMP) Phase 2b study in men who have sex with men, transgender people, and high‐risk heterosexual women, the first study to evaluate the efficacy of VRC01 in reducing sexual transmission of HIV.^9^ AMP demonstrated that a single bNAb, despite good potency and breadth profiles, is insufficient to prevent sexual transmission, but does so (with 75% efficacy) when exposed to a VRC01 neutralization‐sensitive viral strain.^9^ Due to the global diversity of circulating viral clades, a single efficacy criterion—that is the predicted serum neutralization 80% inhibitory dilution titer (PT80), defined as the ratio of plasma Ab concentration:80% inhibitory dilution titer determined in vitro against a given candidate virus—has been proposed as a proxy for protection to be validated or modified in all efficacy trials and in all target populations.^10^ This extrapolation may be valid for HIV prevention in adults, but prevention of HIV transmission in infants and children may require a different approach.

## 3 THE PARADIGM OF HIV TRANSMISSION THROUGH BREASTFEEDING

We suggest that the stance taken for sexual transmission, a possible “one size fits all” fixed combination option, should not be applied to the prevention of HIV transmission through breast milk for several reasons. The first is strategic: should we not wait for Godot—the demonstration of safety, PK, and efficacy of a definitive fixed combination bNAb in all potential target populations and all age groups—to initiate a public health intervention in newborns and infants? The second is pathophysiological: would a triple bNAb combination be critical to prevent HIV in children exposed to nonsexual routes of transmission? The third is ethical: Is it licit to expose individuals (children or adults) to antibodies with low breadth against strains circulating in their region?

It is now widely accepted that at least half of the 130,000 new pediatric HIV infections that had occurred in 2022 on the African continent resulted from breastfeeding transmission.^11^ The ambitious goal of eliminating new pediatric HIV infections by 2030 requires accelerated prevention strategies in high‐risk settings.^12^ In such a context, lessons learned from adults included in the AMP trial may have limited relevance in predicting efficacy in neonates and infants. This population is different—in terms of ontogeny of the immune response, volume of distribution, and PK/PD characteristics, neonates are very different from adults—and the route of transmission involves different tegument interfaces, inoculum, and submucosal environments. Neonates are not miniaturized adults. Unlike adult pre‐exposure prophylaxis (PrEP), which is based on a two‐drug regimen, extended postnatal prophylaxis (PNP) is most often based on a single drug, as combinations have not been shown to be more effective.^13^, ^14^ In addition, the plasma antiretroviral level required to protect against HIV acquisition through breastfeeding in infants is much lower than the therapeutic level.^15^

The mechanisms of transmission differ in many ways between breastfeeding in infants and sexual transmission in adults or adolescents. Some HIV transmission through breast milk may result from cell‐associated HIV^16^, ^17^ involving cell types that have never been documented in sexual transmission. HIV transmission through breastfeeding results from intermittent but repeated exposure (viral shedding in breast milk) to a continuous source of virus (HIV‐infected mother), which is not necessarily the case with repeated sexual HIV exposure in sexual transmission. Finally, both the source of exposure (breast milk) and the exposed individual (breastfed infant) contain high titers of maternal antibodies, a large proportion of which have a repertoire directed toward recapitulation of past and present maternal HIV quasispecies.

## 4 HIV TRANSMISSION THROUGH BREASTFEEDING IN HIGH HIV INCIDENCE AREAS: THE BEST INDICATION FOR PASSIVE IMMUNOPROPHYLAXIS WITH bNAbs?

Consider a hypothetical community with high HIV prevalence and incidence, such as the north‐eastern provinces of South Africa.^18^ In such communities, more than 20% of pregnant women are living with HIV, but prevention of vertical transmission (PMTCT) strategies are successfully implemented, resulting in a residual risk of postnatal transmission of only 0.7% at 10 weeks, and about 1%−2% thereafter.^19^ Babies are breastfed for an average of 6−12 months. In the same community, HIV incidence is particularly high among young women, in the order of 6 per 100 women/year. This epidemiological figure is not uncommon, especially in eastern and southern Africa.^20^ Approximately 30% of breastfeeding women with acute HIV infection transmit HIV to their infants through breastfeeding,^21^ although almost all of them escape the scrutiny of PMTCT programs. Based on these indicators, it can be calculated that for every 10,000 live births, over a 12‐month period there will be 14 HIV transmission events from women living with HIV (women known to be infected before or during their last pregnancy) and 144 events from women having recently contracted HIV (women with acute infection while breastfeeding). In other words, in these communities with high HIV incidence and where the majority of HIV infections acquired in infancy are due to breastfeeding transmission, 90% of new infant infections occur in infants born to mothers with a recent infection, and it is riskier for a newborn to be born to an HIV‐uninfected mother than to be born to a mother living with HIV and virally suppressed on antiretroviral therapy. In HIV infections acquired by breastfeeding from a mother with acute infection, although the inoculum is likely to be high (high maternal viral load), the diversity of transmitted viral variants is thought to be restricted, and transmission results from a narrow, poorly diverse transmitted/founder (T/F) virus population^22^, ^23^ that stochastically falls within the neutralization‐sensitive viruses included in the breadth of appropriately selected bNAbs.^23^ In such a high HIV prevalence and incidence community, the implementation of a passive immunoprophylaxis program administered to all newborns (regardless of HIV exposure status) to prevent postnatal HIV transmission through breastfeeding could be groundbreaking.^12^

The chosen bNAbs would need to be safe and have a favorable PK/PD profile in neonates and infants^4^ and have preferential effector functions against free viral particles and cell‐associated viruses (including antibody‐dependent complement cytotoxicity and/or enhancement of phagocytosis).^24^, ^25^ It is therefore likely that administration to all newborns and breastfed infants of a single or dual bNAb regimen (preferentially targeting different Env epitopes) with 98% breadth against clade C (the virus circulating in southern Africa), such as VRC07‐523LS and CAP256V2LS, may tentatively be highly effective in protecting against HIV transmission through breastfeeding in communities with high HIV incidence. This strategy, which complements current PMTCT initiated during pregnancy (with maternal antiretroviral therapy and PNP), has a high chance of being a game changer in the elimination of pediatric HIV.^12^

## 5 CONCLUSION—LET US GIVE A CHANCE TO PEDIATRIC EXCEPTIONALISM

Once bNAbs candidates are selected, we propose that the first step should be an accelerated Phase 2b‐Phase 3 efficacy trial of universal single (with exceptional breadth) or, more likely, dual bNAbs administration to newborns in high‐incidence settings. This trial should be preceded by careful immunological and virological assessments of T/F viruses using next‐generation sequencing technologies to predict in vivo neutralization sensitivity.^26^ In addition, the neutralization sensitivity of the HIV strains circulating in the target population should also be characterized.

If effective, the potential major advantage of this simple single or dual bNAbs strategy could be local production at lower cost—provided that a program and associated production pathway can be initiated for rapid, immediate implementation in national or regional guidelines. This could be done without waiting for the results of trials evaluating strategies based on fixed combination bNAbs or tri‐ or quadrivalent products.

The history of vaccinology has shown us that pragmatic “test and try” approaches are often successful when supported by strong evidence‐based hypotheses. In high HIV prevalence/incidence settings, we should seize this early opportunity to test simple, population‐targeted bNAb strategies to eliminate HIV transmission through breast milk, rather than waiting for bNAb strategies with universal clade coverage.

## AUTHOR CONTRIBUTIONS

**Philippe Van de Perre**: Conceptualization; validation; writing—original draft; writing—review and editing. **Gabriella Scarlatti**: Validation; writing—review and editing. **Penny L. Moore**: Validation; writing—review and editing. **Jean‐Pierre Molès**: Validation; writing—review and editing. **Nicolas Nagot**: Validation; writing—review and editing. **Thorkild Tylleskär**: Validation; writing—review and editing. **Glenda Gray**: Validation; writing—review and editing. **Ameena Goga**: Conceptualization; validation; writing—original draft; writing—review and editing.
